# 2024 Jack Kenney Award for Outstanding Service

**DOI:** 10.1128/jb.00494-24

**Published:** 2025-01-31

**Authors:** George A. O’Toole

**Affiliations:** 1 Department of Microbiology and Immunology, Geisel School of Medicine at Dartmouth 12285 , Hanover, New Hampshire, USA

**Keywords:** Kenney Award, reviewer, acknowledgment

## EDITORIAL

Since 2009 we have conferred the Jack Kenney Award for Outstanding Service to a reviewer who does extraordinary service for the journal. I had the opportunity to work with Jack as an editor, including on my first special meeting collection that he helped me put together. Jack was an outstanding production editor who retired in 2010; he died shortly after his retirement.

I am pleased to announce that Melanie Barnett is the 2024 recipient of this award. Dr. Barnett is pictured here with her award.


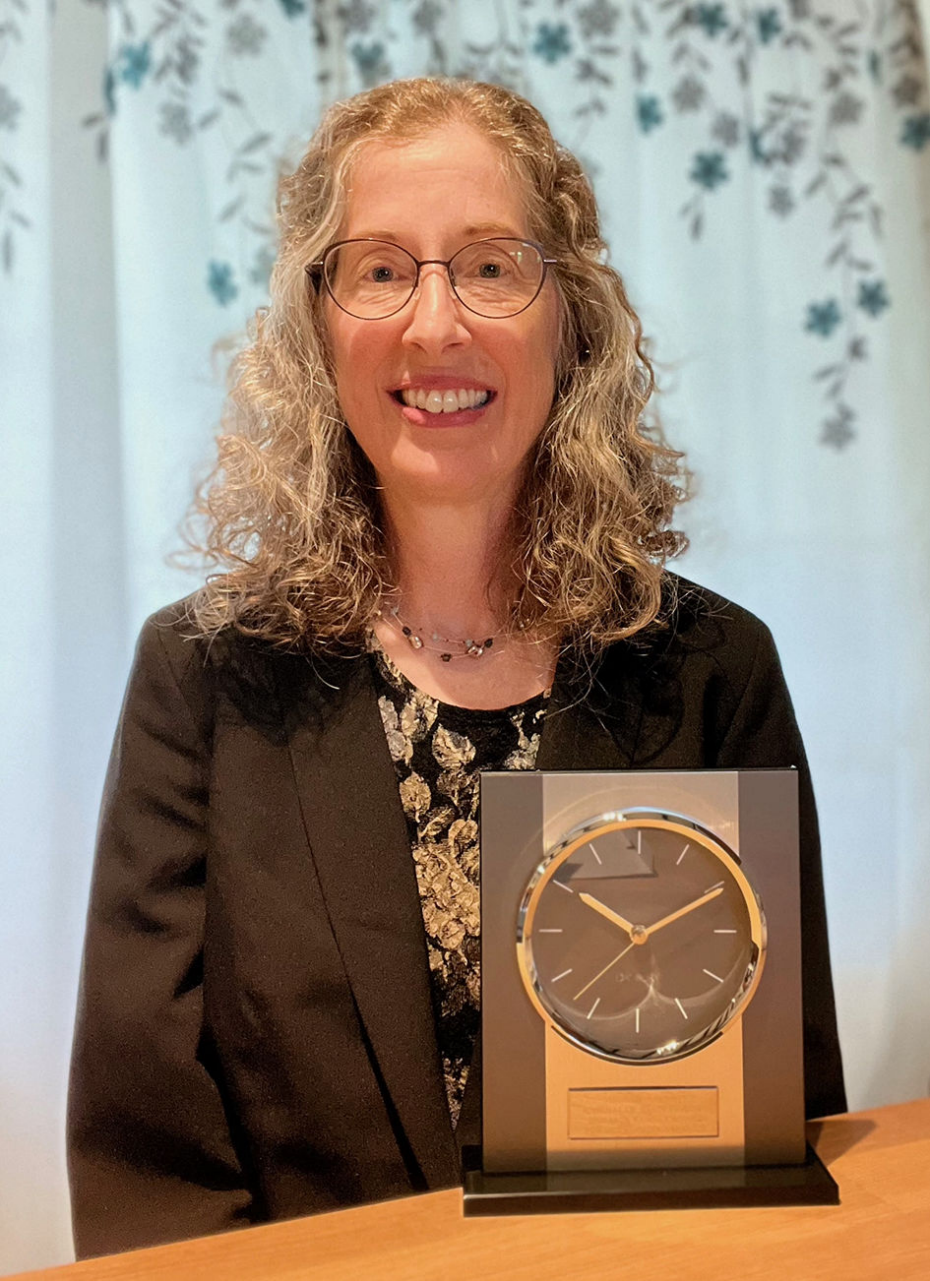


Dr. Barnett is an Emerita Senior Research Scientist from the lab of Dr. Sharon Long at Stanford University. The Long lab studies the soil-dwelling, symbiotic, nitrogen-fixing alphaproteobacterium *Sinorhizobium meliloti,* in particular, its interaction with leguminous host plants and its stress response mechanisms. During her many years in the Long lab, Dr. Barnett’s research projects included the characterization of bacterial symbiotic and stress response genes and regulatory circuits, as well as *S. meliloti* genomics.

Over the past year, Dr. Barnett reviewed five manuscripts for JB—she never declined a request, and her feedback was considered outstanding by the editors.

The editors are pleased to recognize Dr. Barnett’s outstanding service to JB and society publishing.

